# Interactions between density, home range behaviors, and contact rates in the Channel Island fox (*Urocyon littoralis*)

**DOI:** 10.1002/ece3.1533

**Published:** 2015-05-29

**Authors:** Jessica N Sanchez, Brian R Hudgens

**Affiliations:** 1 Institute for Wildlife StudiesArcata, California, USA; 2 School of Veterinary Medicine, University of CaliforniaDavis, California, USA

**Keywords:** Contact, density dependence, disease transmission, home range, intraspecific competition, proximity logger, spatial ecology, territoriality

## Abstract

Many of the mechanisms underlying density-dependent regulation of populations, including contest competition and disease spread, depend on contact among neighboring animals. Understanding how variation in population density influences the frequency of contact among neighboring animals is therefore an important aspect to understanding the mechanisms underlying, and ecological consequences of, density-dependent regulation. However, contact rates are difficult to measure in the field and may be influenced by density through multiple pathways. This study explored how local density affects contact rates among Channel Island foxes (*Urocyon littoralis*) through two pathways: changes in home range size and changes in home range overlap. We tracked 40 radio-collared foxes at four sites on San Clemente Island, California. Fox densities at the four sites ranged from 2.8 ± 1.28 to 42.8 ± 9.43 foxes/km^2^. Higher fox densities were correlated with smaller home ranges (*R*^2^ = 0.526, *F*_1,38_ = 42.19, *P *<* *0.001). Thirty foxes wore collars that also contained proximity loggers, which recorded the time and duration of occasions when collared foxes were within 5 m of one another. Contact rates between neighboring fox dyads were positively correlated with home range overlap (*R*^*2*^ = 0.341, *P *=* *0.008), but not fox density (*R*^*2*^ = 0.012, *P *=* *0.976). Individuals at high densities had more collared neighbors with overlapping home ranges (*R*^*2*^ = 0.123, *P *=* *0.026) but not an increase in the amount of contact between individual neighbors. This study was the first time contact rates were directly measured and compared to density and home range overlap. Results suggest that foxes exhibit a threshold in their degree of tolerance for neighbors, overlap is a reliable index of the amount of direct contact between island foxes, and disease transmission rates will likely scale with fox density.

## Introduction

Close contacts among individuals influence the spread of directly transmitted pathogens (Keeling 1999; Altizer et al. 2003; Böhm et al. 2009; Cross et al. 2009; Hamede et al. 2009), competition and predation risk (Mills and Gorman 1997; Berger and Gese 2007), resource utilization and habitat use among species (Major and Sherburne 1987; Weissinger et al. 2009), determine the organization of social groups and mating patterns (Tucker et al. 1993; Ramsey et al. 2002), and explain reproductive phenomena (Ordiz et al. 2008). However, measuring the frequency and duration of close contact in free-ranging wildlife is difficult, especially for species that are nocturnal, cryptic, or otherwise difficult to observe (Ji et al. 2005; Prange et al. 2006).

Contacts among individuals or groups are likely to be influenced by various aspects of a species’ ecology, such as the degree to which territories or areas of exclusive use are maintained, the number of individuals with neighboring (adjacent) home ranges, and the amount of contact those neighbors have with one another (Woodroffe 1999). These factors in turn depend on the density of the population, which can influence contact rates by altering home range sizes and the amount of overlap between home ranges (McCallum et al. 2001). However, these relationships are not always intuitive or straightforward. For example, badger (*Meles meles*) culling to lower densities in an effort to control the spread of bovine tuberculosis (*Mycobacterium bovis*) led to increased movement between social groups (Tuyttens et al. 2000) and a greater number of overlapping ranges, but no change in the proportion of each home range that overlapped with other ranges (Woodroffe et al. 2006). As a consequence of these social disturbances, the incidence of bovine tuberculosis increased at study sites with repeated badger culling, despite the generally accepted theory that lower host densities reduce host contact rates and result in lower rates of disease transmission (Woodroffe et al. 2006).

**Figure 1:**
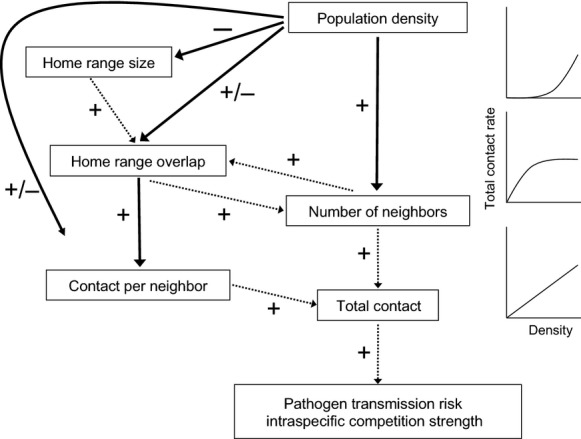
Possible pathways through which increases in density might influence contact rates, leading to accelerating, saturating, or linear relationships between density and contact rates. Solid lines indicate hypothesized pathways tested in this study. Dashed lines indicate pathways not directly addressed in this study.

Changes in density may also directly influence contact rates independent of changes in home range overlap. For example, male brushtail possums (*Trichosurus vulpecula***)** responded to decreases in relative density by expanding their home ranges and overlap with females so that male–female contact rates increased but male–male contact rates decreased, indicating that males were altering their home ranges to maintain their access to females (Ramsey et al. 2002). Conversely, North American red squirrels (*Tamiasciurus hudsonicus*) responded to increases in density by decreasing their direct contacts through antagonistic physical interactions (Dantzer et al. 2012). Understanding the influence of population density on contact rates, and the pathways by which this influence is exerted (Fig.1), would be extremely useful in anticipating contact rates among individuals in populations with heterogeneous spatial distributions.

Home range overlap among individuals has been examined across seasons, sexes, and even species within a study site (Hill and Lein 1989; Horner and Powell 1990; Drygala et al. 2008; Harrington and Macdonald 2008), but few studies have compared overlap at different local densities of the same species (Woodroffe et al. 2006; Guyer et al. 2012). Although poorly understood, the relationship between density and overlap can have significant implications for ecological relationships mediated through contact rates among neighbors.

**Figure 2:**
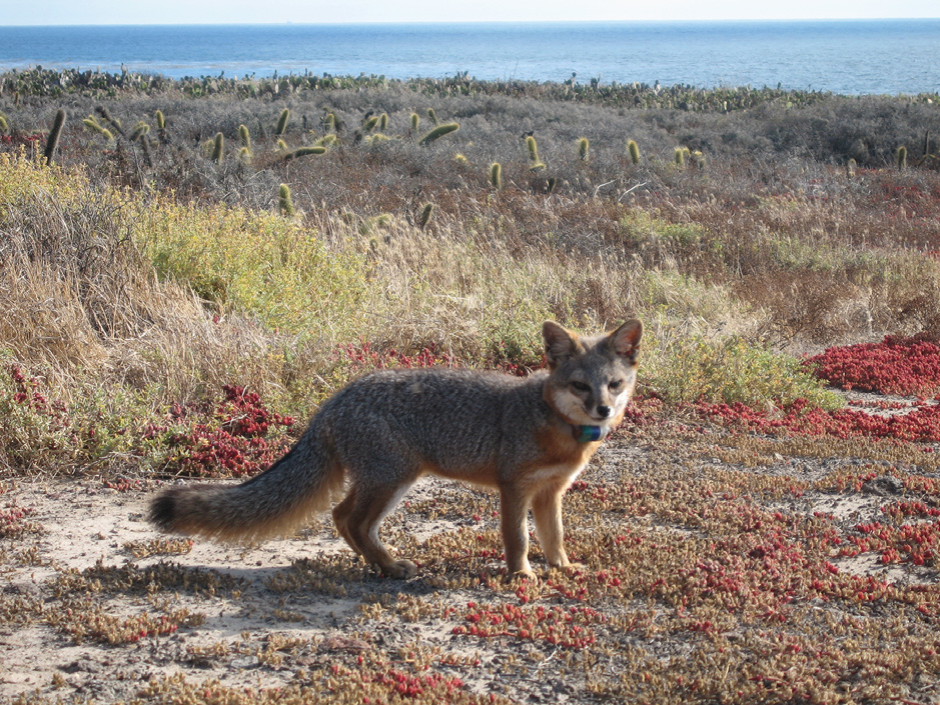
Radio-collared Channel Island fox (*Urocyon littoralis*) on San Clemente Island, California (Photo: J. N. Sanchez).

This study explored how population density affects individual contact rates through variations in home range size and overlap in the Channel Island fox (*Urocyon littoralis*) on San Clemente Island (SCI), California (Fig.2). This study was motivated by concerns about how best to manage disease risk for canine distemper virus (CDV) after an 85% decline of the closely related Channel Island fox population on Santa Catalina Island, California, due to a CDV epidemic (Timm et al. 2009; Munson 2010). The island fox also has several ecological and life-history characteristics that make it an ideal system to examine the relationships between density, home range behaviors, and contact rates. Island fox densities vary widely over relatively small spatial scales (Coonan 2011), allowing us to measure home ranges and contact rates at a range of densities within the same population. In addition, SCI has only one sympatric carnivore species (introduced feral cats; *Felis catus*; Coonan 2010) that might influence fox home ranges, compared to mainland wildlife populations where numerous species may utilize similar habitats and resources.

Island foxes form socially monogamous pairs that occupy the same home range year round and remain together unless one member of the pair dies (Roemer et al. 2001). Home range overlap is substantially greater between mated pairs than between unmated pairs (Crooks and van Vuren 1996; Roemer et al. 2001), and adult offspring may establish home ranges that overlap extensively with their parents (Roemer et al. 2001). Previous work has shown that island fox home range size and core areas do not differ significantly among seasons (Crooks and van Vuren 1996) or between males and females (Crooks and van Vuren 1996; Roemer et al. 2001).

## Materials and Methods

San Clemente Island is owned by the United States Navy and is located approximately 109 km west of San Diego, California. It has an area of 146 km^2^, tapering from 6.4 km wide at its southern end to 2.4 km wide at its northern end. Most of the island is a semi-flat plateau with steep cliffs on the eastern edge, and gradually declining marine terraces on the western side. The highest elevation is 559 m above sea level. Average annual precipitation is 13.2 cm (Olsen et al. 2000), and the dominant habitat types include grassland, maritime desert scrub, and coastal dunes (Jorgensen and Ferguson 1984). Maritime desert scrub (MDS) habitat can be further separated into areas dominated by California boxthorn (*Lycium californicum*) generally corresponding to where the terrain consists of gently sloping marine terraces, and prickly pear (*Opuntia littoralis*) generally corresponding to where there are steep, rocky canyons. For purposes of this study, these habitats are referred to as “MDS gentle” and “MDS rugged” reflecting postulated potential influences of topography on fox densities.

Foxes were radio-tracked in four areas on SCI to test for differences in density that may affect both the number and frequency of contacts among animals with neighboring home ranges. A subset of foxes at each site wore radio collars also containing proximity loggers that recorded data about close contacts among individuals. These contacts were used to determine how density-mediated changes in home range behaviors affected contact rates between individuals.

Foxes were trapped from July to August 2010 to apply collars containing radio transmitters and to assess fox densities. The four trapping grids where foxes were radio-collared were each contained in four of the dominant habitat types. Grid 1 was located within the sand dune habitat and adjacent to gentle maritime desert scrub on the northern end of SCI. Grid 2 was located within gentle maritime desert scrub on the western coast next to the shoreline. Grid 3 was located in rugged maritime desert scrub habitat adjacent to grasslands on a mid-level marine terrace on the western slope of the island. Grid 4 was located in grassland habitat adjacent to rugged maritime desert scrub habitat on the upper plateau.

Traps were set 250 m apart in five trap × eight trap grids (1 km × 1.75 km) and were run for a minimum of four nights to enable the calculation of density estimates for each study site. In order to fit Grid 1 entirely within the sand dunes habitat, it was reduced in size to a four trap × eight trap grid (0.75 km × 1.75 km). Traps were run for up to 7 days, if needed, to collar the target number of animals. Foxes were caught using box traps (23 × 23 × 66 cm; Tomahawk Live Trap Co., Tomahawk, WI) covered with burlap and vegetation to provide protection from the elements, and lined on the inside with grass as bedding material. Traps were baited using dry cat kibble and berry-scented lure (Knobb Mountain Fur Company, Berwick, PA). “Bite bars” made of polypropylene tubing attached to the inside of the trap with flexible wire were added to each trap for foxes to release stress without damaging their teeth. For each animal captured the date, time, trap location, passive integrated transponder (PIT) tag ID, sex, and age class were recorded. A subcutaneous PIT tag was inserted between and just cranial to the scapulae if an animal had not been previously tagged.

At each site, eight foxes (four males and four females) were fitted with proximity collars containing both UHF proximity loggers and VHF transmitters (Sirtrack Limited, Havelock North, New Zealand). An additional 10 collars containing only VHF transmitters (Communication Specialists, CA) were distributed at two sites to ensure a representative number of foxes were collared at each site. We placed an additional eight collars at Grid 1 due to the extremely high density of foxes captured there during initial trapping, and two additional collars were placed on foxes opportunistically at Grid 4. Both collar types weighed approximately 40 g, which was no more than 3% of an animal's body weight. Adult foxes in good health were preferentially collared, as they were the least likely to disperse from their home range or die during the 6-month anticipated battery life of the radio transmitters. The foxes chosen to be collared were caught in traps that were immediately adjacent to one another or as close as possible to maximize the likelihood that foxes had “neighboring” home ranges.

Collared foxes were tracked via radiotelemetry from July 2010 to February 2011. Each fox was located one to two times per week over the 6-month study period. Nocturnal locations were not collected due to safety restrictions, but foxes were monitored as evenly as possible between dawn and dusk. Because island foxes are active throughout the day and night, and crepuscular activity levels are similar to nocturnal activity levels (Laughrin 1977; Crooks and van Vuren 1995; Hudgens and Garcelon 2011) the lack of nocturnal locations should not have biased results. Triangulations consisted of ≥3 bearings, 30° to 150° apart, taken within 20 min of each other. Fox location estimates were generated from triangulations in the program Location of a Signal (“LOAS;” Ecological Software Solutions, CA) using the maximum likelihood estimator. Telemetry error was estimated for each habitat type by triangulating test collars placed within each site at multiple locations unknown to the tracker, and using LOAS to estimate the standard deviations of bearing error from the true collar location. Test collar locations were confirmed using a handheld global positioning system with 5 m accuracy. These standard deviations were used in the calculations of error ellipses around fox locations within LOAS.

The detection distance of the proximity collars was set to approximately 5 m, which we estimated to be a reasonable distance from which two animals could detect one another (White and Harris 1994) or for infectious disease to be transmitted via aerosol (Gorham and Brandly 1953; Xie et al. 2007). The true detection distance of the collars varied due to terrain, the absorption of radio signal by the animal's body, and the orientation of each animal to one another (Sirtrack Limited 2008). The average collar detection distance was tested by pairing collars at random, placing them on the carcasses of road killed foxes, and slowly moving one fox toward the other along a measuring tape from 0 to 15 m while recording the time at 0.3 m intervals. Data were downloaded from both collars and the time stamp when each collar first detected the other was matched with the distance apart the collars were at that time, and this distance was averaged across all collars. A contact record was programmed to end when the foxes were separated by at least 5 m for 120 sec. For each contact, proximity collars recorded the collar ID of the other fox, the date and time the contact began, and the duration of the contact in seconds. Collars were removed, and the contact data were downloaded between December 2010 and January 2011.

Density estimates for each site were calculated from mark–recapture data collected during the first 4 days of trapping using analyses of spatially explicit capture–recapture data developed by Efford et al. (2004) as implemented in Program DENSITY (University of Otago, Otago, New Zealand). Each trapping grid was almost completely contained within a single habitat, and thus, density estimates from the trapping grids were used to assign a fox density value to each habitat type.

Ninety-five percent fixed kernel home ranges were calculated for each fox, using locations collected over the life of the collar, to control for locations that might represent movements outside the true home range (Okarma et al. 1998; Dickson and Beier 2002). Fox locations were entered into Program R (R Foundation for Statistical Computing, Vienna, Austria, http://www.R-project.org), and the so-called plug-in method (Duong 2007) was used within the package “ks” to calculate the kernel bandwidths for each fox and generate utilization distributions for each home range. Hawth's Tools (Spatial Ecology LLC, http://www.spatialecology.com/htools) extension for ArcMap 9.3 was used to generate 95% volume contours from the utilization distributions, and Geospatial Modelling Environment (GME; Spatial Ecology LLC, http://www.spatialecology.com/gme) was used to determine the area of overlap between each pair of home ranges. Two-dimensional home range overlap between each fox dyad was determined by calculating the geometric mean of the ratio of overlap area to the total home range area of each fox (Minta 1992).

A post hoc analysis was performed to test for biases in home range size due to the number of locations obtained for each animal. An area-observation curve was generated for each fox by plotting the number of locations (from three to the maximum number obtained for each individual) against the corresponding minimum convex polygon home range size (Odum and Kuenzler 1955). We deemed that a sufficient number of locations had been collected to accurately represent a home range if the area-observation curve for that individual had begun to asymptote before the maximum number of locations taken for that animal.

For each fox dyad, the total number of contacts and duration of time in contact over the life of both collars were used to calculate the number of contacts per day and seconds in contact per day. Both collars in a dyad recorded contacts between foxes, and often these records differed slightly between collars due to differences in transmitter strength, receiver sensitivity, and the location of each animal in the environment during a contact. To account for these differences within each fox dyad, contact records that temporally overlapped were merged, resulting in a record that reflected the maximum duration of each contact.

“Neighbors” were defined as foxes with 95% fixed kernel home ranges that overlapped or had borders ≤250 m of one another, representing between 25% and 50% of the mean maximum distance moved between traps overlapping each area during a population monitoring study run from 2001 to 2006 (D. Garcelon, Institute for Wildlife Studies, unpubl. data). This threshold was chosen because it was approximately half the radius of the previously reported average SCI fox home range size (Spencer et al. 2006; Resnik 2012). Foxes with measured home ranges that were near one another but did not overlap still had the potential to encounter one another while maintaining home range boundaries or during occasional forays outside their primary home range areas (White and Harris 1994). However, foxes generally do not travel long distances outside their home range (Roemer et al. 2001), making it unlikely that two foxes occupying home ranges separated by a large distance would encounter one another. The ≤250 m neighbor threshold was implemented to include nonoverlapping neighbors in analyses of how overlap relates to contact, while also avoiding skewing the data by including fox dyads that were not actually neighbors because they were so far apart they never had the chance to encounter one another. Because some home ranges in the highest density site were <250 m across (see Results), all analyses were repeated including only fox dyads that had overlapping 95% fixed kernel home ranges or contacts recorded by the proximity loggers, to test for any biases introduced by including nonoverlapping neighbors. There was no difference in the results of analyses with or without the 250 m buffer, except as noted below.

Although trapping grids were restricted to single habitats, some collared foxes had home ranges that extended into adjacent habitats. To account for this, each fox was assigned a weighted density value based on the percentage of its home range area that occupied each habitat type. This weighted density value was then used in regressions with home range size and the number of neighboring foxes with overlapping home ranges. Only overlapping home ranges were counted when determining the number of neighbors to regress against density, ensuring a conservative estimate of the relationship between these variables. The individual weighted density values of both foxes in each dyad were averaged for regressions of density with home range overlap and contact rates (where each data point represents two individuals). The amount of home range overlap and contact rates between each pair of neighboring foxes were also regressed. If the variance of any of these variables scaled with the mean, they were log-transformed if the datasets did not contain zeros, or square-root-transformed if the dataset contained zeros. Because home range overlap and pairwise contact data included nonindependent data points from each fox, regressions including these parameters were bootstrapped to determine statistical significance.

In order to minimize the confounding effect individual fox relationships might have had on analyses, we separated data from fox dyads that were determined be mates or family members from unrelated neighboring fox dyads in analyses. Mates and family members were excluded from analyses because they are known to have much higher home range overlap (Crooks and van Vuren 1996) and orders of magnitude higher contact rates (Ralls et al. 2013) than nonrelated dyads regardless of the local fox density, and there were too few mated pairs to conduct separate statistical analyses. Because the difference in the contact rates between related and nonrelated dyads was so large, combining mates and unrelated neighbors in analyses could have obscured differences among sites and any influence density had on fox behavior. Fox dyads were determined to be mates or family members if they satisfied at least two of the following criteria: they had a large amount of home range overlap (>50%), were visually observed resting or foraging together, or had contact rates ≥2 times greater than nonrelated dyads (Ralls et al. 2013).

## Results

The number of foxes captured on each grid ranged from 12 to 45. There was approximately a 15-fold difference in fox densities between grassland and dune habitats while the two maritime desert scrub habitats supported intermediate densities of foxes (Table1). In addition to the four females and four males fitted with proximity collars at each site, eight foxes (seven females and one male) at Grid 1 and two foxes (both males) at Grid 4 were fitted with VHF only collars in an effort to standardize the proportion of animals collared at each site.

**Table 1:** Results of grid trapping for foxes on San Clemente Island, California, in July 2010

Grid number	Dominant habitat	Neighboring habitat	Individuals caught	Number of foxes collared	Density (foxes/km^2^) 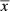 ± SE
1	Sand dune	Gentle MDS	45	16	42.6 **±** 9.4
2	Gentle MDS	Gentle MDS	15	8	4.1 **±** 1.8
3	Rugged MDS	Grassland	21	8	11.3 **±** 4.7
4	Grassland	Rugged MDS	12	10	2.9 **±** 1.3

MDS, maritime desert scrub.

Of the 42 foxes collared, two foxes wearing proximity collars from Grid 4 were dropped from analyses. One male was never relocated after being collared, and it was believed his VHF transmitter failed. One female was located throughout the study period, but was found dead at the end of the season. She was removed from home range analyses because her locations for the previous months were very close to one another, indicating that she may have been sick or injured and not behaving normally for some time before her death. The remaining 40 foxes were relocated a total of 11–30 times each (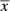 = 20.0 ± 0.8).

**Figure 3:**
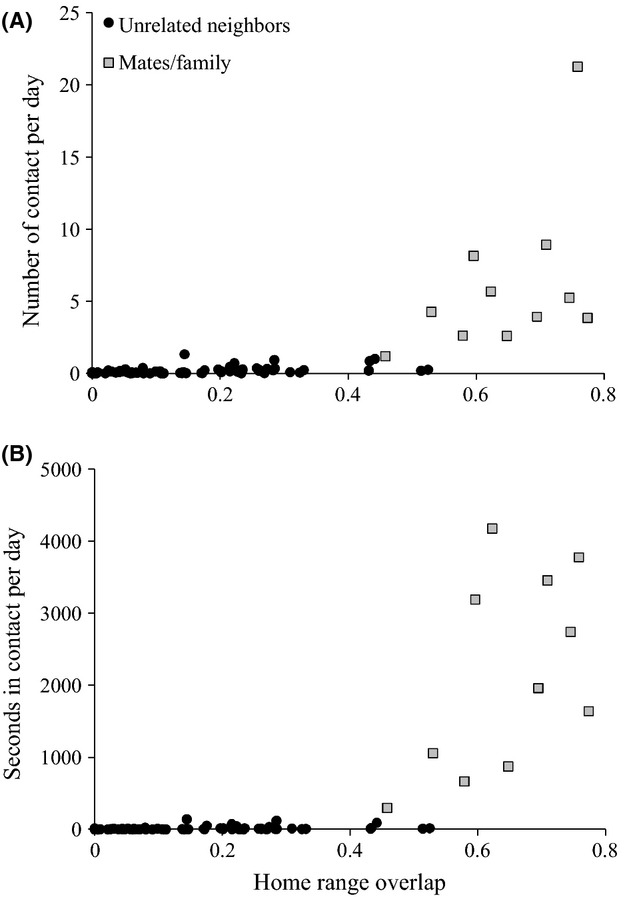
Comparisons of the proportion of home range overlap with the number of contacts per day (A) and seconds in contacts per day (B) between each unrelated fox dyad (circles) and mated pairs or family dyads (squares).

Mated pairs and related animals (*n* = 11) were inferred by considering the amount of home range overlap between each female–male dyad (>50%) and records of the dyad being visually observed resting or foraging together. When we compared contact rates among each fox dyad to the amount of overlap between their home ranges, we observed a break point where some dyads had contact rates ≥2 times higher than others for a similar degree of overlap (Fig.3). We chose this empirical break point as our cutoff for categorizing dyads as related (mates and family members) and nonrelated for further analyses.

The average detection distance of the proximity collars was 4.6 m (*n *=* *61, SD = 1.3 m). Proximity collars lasted an average of 76.5 days (SD = 13.2). At each study site, 8–12 test collars were hidden and triangulated to determine the bearing error for each study site (Table2).

**Table 2:** Mean (±SE) minimum convex polygon (100% MCP) and fixed kernel (50%, 85%, and 95% FK) estimates of island foxes home range sizes (km^2^) and results of telemetry error testing on San Clemente Island, California, between July 2010 and February 2011

Dominant habitat	Number of foxes collared	Mean (±SE) home range size	Mean area of error ellipse around triangulated fox locations (km^2^ ± SE)	Total number of test collars triangulated	Average SD of bearing error for test collars
Sand dune	12	0.21 (0.05)	0.01 (0.0002)	9	10.6
Gentle MDS	12	0.87 (0.14)	0.007 (0.0003)	10	7.5
Rugged MDS	9	0.99 (0.18)	0.007 (0.0002)	12	10.8
Grassland	7	1.39 (0.27)	0.02 (0.0004)	8	11.8

MDS, maritime desert scrub.

**Figure 4:**
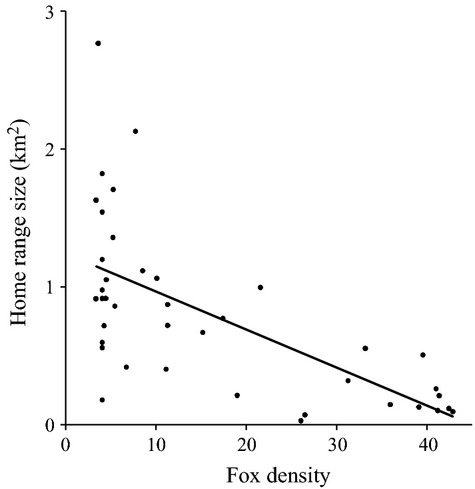
Comparisons of San Clemente Island fox density with home range size (natural log-transformed, *R*^2^ = 0.526, *F*_1,38_ = 42.19, *P *<* *0.001).

Ninety-five percent FK home range sizes did not differ between females (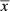 = 0.69 km, SE = 0.09 km) and males (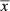 = 0.92 km, SE = 0.19 km; *P *=* *0.26). Home range size was negatively correlated with local fox density (natural log transformed; *R*^2^ = 0.526, *F*_1,38_ = 42.19, *P *<* *0.001; Fig.4).

**Figure 5:**
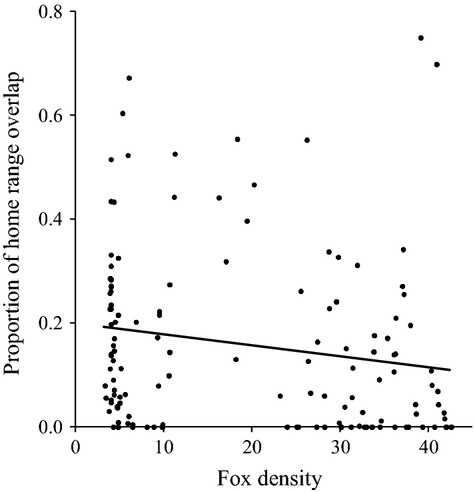
Comparisons of San Clemente Island fox density with the proportion of home range overlap between each fox dyad (square-root transformed, *R*^*2*^ = 0.032, *P *=* *0.04).

Fox density had a slight negative correlation with the proportion of home range overlap (square-root transformed; *R*^*2*^ = 0.032, *P *=* *0.04; Fig.5), which was not apparent when only neighbors with overlapping 95% fixed kernel home ranges were considered (*R*^*2*^ = 0.01, *P *=* *0.512). There was a weak but positive relationship between local fox density and the number of overlapping home ranges with other collared animals (*R*^*2*^ = 0.123, *P *=* *0.026).

**Figure 6:**
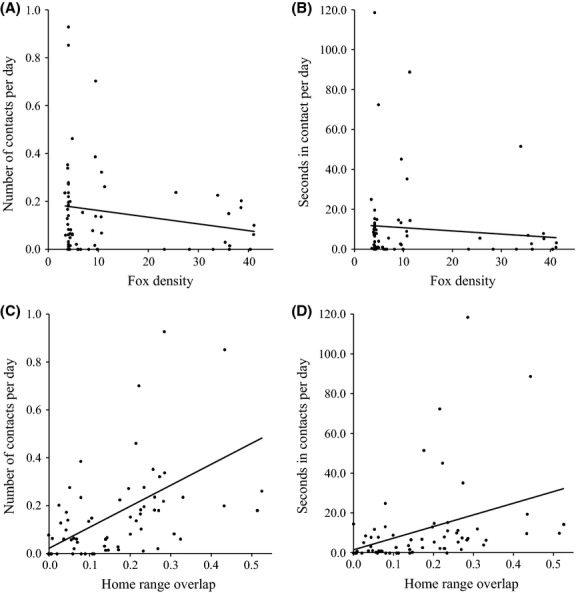
Comparisons of San Clemente Island fox density with the number of contacts per day between each unrelated fox dyad (A; square-root transformed, *R*^*2*^ = 0.012, *P *=* *0.976), and seconds in contacts per day between each unrelated fox dyad (B; square-root transformed, *R*^*2*^ = 0.003, *P *=* *0.998). Comparisons of the proportion of home range overlap with the number of contacts per day between each unrelated fox dyad (C; square-root transformed, *R*^*2*^ = 0.341, *P *=* *0.008), and seconds in contacts per day between each unrelated fox dyad (D; square-root transformed, *R*^*2*^ = 0.229, *P *=* *0.014).

For each fox dyad, neither the number of contacts per day (square-root transformed; *R*^*2*^ = 0.012, *P *=* *0.976; Fig.6A) nor the seconds in contact per day (square-root transformed; *R*^*2*^ = 0.003, *P *=* *0.998; Fig.6B) was correlated with the local density of foxes. However, home range overlap was positively correlated with both the number of contacts per day (square-root transformed; *R*^*2*^ = 0.341, *P *=* *0.008; Fig.6C) and seconds in contact per day (square-root transformed; *R*^*2*^ = 0.229, *P *=* *0.014; Fig.6D) between fox dyads.

## Discussion

**Figure 7:**
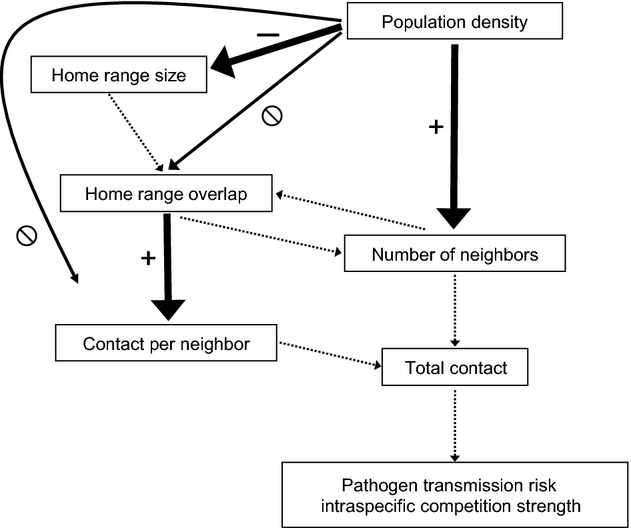
Pathways through which increases in density influence contact rates, as determined by this study. Thick solid lines indicate hypothesized pathways supported in this study. Thin solid lines indicate hypothesized pathways not supported in this study. Dashed lines indicate pathways not directly addressed in this study.

Our study confirmed two intuitive patterns of home range behaviors: local fox density was negatively correlated with home range size, and home range overlap was positively correlated with contact rates (Fig.7). We also found that home range overlap and contact rates among unrelated neighboring dyads were not correlated with local fox density, but there was a weak positive relationship between density and the number of collared neighbors with overlapping home ranges (Fig.7). Consequently, contact rates increased linearly with density due solely to an increase in the number of collared neighbors. For each fox dyad, the association between home range overlap and contact rates was heavily influenced by the social relationship between the two foxes, with mates and family members having much higher rates of contact with one another than unrelated dyads.

Measuring the true number of neighbors each fox had was not a primary goal of this study, and we were only able to measure the number of collared neighbors that interacted with one another. Biases may have been introduced to data from the high-density site (Grid 1) where a lower proportion of foxes were collared (36% of captured foxes at the highest density site vs. 83% at lowest density site) despite nearly twice as many foxes being collared compared to any other site. The unexpectedly small home ranges of foxes at the highest density site likely introduced additional biases due to the intertrap distance being approximately equal to the radius of the average home range (if we assumed circular home ranges). At all other sites, the same intertrap distance represents only approximately half of a home range radius, making it much more likely that a fox with a home range centered within the trapping grid would have at least one trap within its home range and be captured and collared. As a consequence, our estimates of the number of overlapping home ranges each fox has at high densities were biased low. However, despite this bias, we still detected a significant positive relationship between fox density and the number of neighbors, suggesting that the true relationship is much stronger than we were able to measure.

Establishing a distance threshold of home range borders within which animals are considered neighbors is also problematic, as interactions among neighbors with nonoverlapping borders (e.g., during exploratory forays) are less likely if the intervening space represents a large fraction of another animal's home range. At the high-density site where home ranges were relatively small compared to intertrap distances, there was a reduced probability of collaring two animals with intermediate Minta overlap values. The direction and degree of bias is not intuitively clear but could be determined through simulation. However, we suspect that these potential biases had little impact on our results as we observed similar patterns when Grid 1 was excluded. Across an approximately fourfold difference in fox densities among the remaining grids, home range size decreased with fox density, and there was little influence of density on home range overlap or contact rates among unrelated fox dyads.

Even at relatively low densities, island foxes exhibited a high tolerance for home range overlap with unrelated neighbors (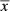 = 20.0%, SD = 12.6%). This finding is consistent with other island species, which typically occur at higher densities and have an increased tolerance for home range overlap with neighbors and acceptance of subordinates, and reduced aggression toward conspecifics compared to their mainland counterparts (Stamps and Buechner 1985). High overlap tolerance among neighbors may be further encouraged by short dispersal distances, often resulting in adult offspring establishing home ranges adjacent to, or overlapping with, their parents’ home ranges (Roemer et al. 2001). However, island foxes do appear to have a threshold for the amount of overlap they will tolerate with neighbors that does not dissolve even at extremely high densities. Similar overlap thresholds have been found across a range of species (Zoellick et al. 2002; Wauters et al. 2005; Wronski 2005; Vashon et al. 2008), but have rarely been measured within the same population at different densities (Hoset et al. 2008). SCI fox home ranges at the highest density site were small enough that the intertrap distance at which animals were collared was larger than the average home range diameter, which may have biased our results toward less observed overlap at that site. Therefore, the total amount of contact a fox experienced with all its neighbors may be larger than we measured at high-density sites, but this appears to be caused by the increase in the number of neighbors and not through density-dependent changes in home range overlap. Similar patterns are observed when Grid 1 is excluded.

It is not always feasible to use methods such as GPS collars or simultaneous triangulations to determine whether neighboring animals are avoiding one another temporally, and in some wildlife studies, the only measure of association available is the spatial overlap of home ranges. The positive relationship between island fox pairwise contact rates and home range overlap supports the methods employed by previous studies, which used overlap as an index of contact among neighbors (Crooks and van Vuren 1996; Roemer et al. 2001; Jiménez 2007, Early 2009). However, this relationship may not hold true for all species. In contrast to island foxes, proximity logger data from European wild rabbits (*Oryctolagus cuniculus*) revealed relatively infrequent contact among individuals of the same group, even among individuals sharing warrens, food resources, and social associates (Marsh et al. 2011). Intergroup contacts were even lower, despite a high percentage of spatial overlap between groups (Marsh et al. 2011). These contrasting results between island foxes and European rabbits demonstrate the importance of measuring contacts directly to fully understand the social behaviors of a species.

One of the most important reasons to better understand contact rates among individuals is to predict the spread of infectious disease in wildlife populations (Altizer et al. 2003; Cross et al. 2009). The patterns of contact among hosts determine whether pathogen transmission will increase linearly with density (density-dependent) or be independent of host density (frequency-dependent; McCallum et al. 2001; Ryder et al. 2007; Smith et al. 2009). Epidemiological models have demonstrated that disease dynamics can differ drastically depending on which type of transmission is used (McCallum et al. 2001; Ryder et al. 2007; Smith et al. 2009). Territorial animals are predicted to display frequency-dependent pathogen transmission when there is an upper limit to the number of neighbors they will encounter (Begon et al. 2002; Smith 2006; Vynnycky 2010). Although island foxes have some degree of restricted overlap between each pair of neighbors, the number of neighbors they contact appears to continue increasing at higher densities. This suggests that pathogen transmission among foxes may be similar to density-dependent models, with transmission increasing linearly as the number of neighboring home ranges increases (White et al. 1995; McCallum et al. 2001; Begon et al. 2002). Such an insight into potential pathogen spread could help mangers prepare for novel disease introduction or epidemic outbreaks, which can be especially devastating for isolated or endangered populations (Woodroffe 1999).

Home ranges are dynamic, and animals may respond to increases in density with compensatory behaviors to reduce their interaction or competition with neighbors. One way animals might mitigate the total negative impact of living in areas with higher density is by reducing home range size such that the number of overlapping home ranges does not scale linearly with density (McLoughlin et al. 2000), and a negative relationship between population density and home range size has been found in a variety of species, including roe deer (*Capreolus capreolus*; Kjellander et al. 2004), red foxes (*Vulpes vulpes*; Trewhella et al. 1988), field voles (*Microtus agrestis*; Erlinge et al. 1990), brown anole lizards (*Anolis sagrei;* Schoener and Schoener 1982), juvenile steelhead trout (*Oncorhynchus mykiss*; Keeley 2000), and breeding European jays (*Garrulus glandarius*; Grahn 1990). While island foxes appear to employ this strategy, home range sizes at high density do not decrease enough to prevent an increase in contact with other foxes. If aggressive interactions carry constant risk of injury or sickness (e.g., as observed with Tasmanian Devil facial cancer; Hamede et al. 2009), aggressive territorial defense with any given neighbor at high densities will carry the same cost as at low densities, but result in a lower net benefit in terms of reducing the proportion of a fox's home range not shared with other unrelated animals.

The contact pattern created by this type of compensatory behavioral response to increased density may obscure disease transmission patterns, making it more difficult to identify so-called super-spreaders (Lloyd-Smith et al. 2005). For example, for two of our mated foxes, contact rates were only 12 times higher than the average contact rate of unrelated pairs. At the lowest density site (Grid 4), where foxes had an average expected 5–6 unrelated neighbors with overlapping home, these mated pairs would come into contact with each other 2 times more frequently than with all other neighbors, but at the two highest density sites, where foxes had an average expected 9–10 unrelated neighbors with overlapping home ranges, these pairs would come into contact with an unrelated neighbor almost as frequently as with each other. Consequently, the larger number of potential pathways for a pathogen to spread from neighbor to neighbor at high densities makes it more difficult to determine the source of any given infection and to identify variation in the contribution of each animal to disease spread.

This study is the first we are aware of that simultaneously measured density, home range behaviors, and close contacts among individuals of the same population. While we verified for island foxes common assumptions that contact rates are positively correlated to both home range overlap and population density, further studies in other populations are needed to generalize the validity of these assumptions and understand when they are likely to be violated. For example, social structure clearly influences the relationship between home range overlap and contact rates (Marsh et al. 2011, this study), but we do not know under which circumstances social structuring obviates or merely obscures density-mediated changes in contact rates. Until we build such an understanding, we should not dismiss the effects of density-mediated differences in contact rates in populations that are highly socially structured. Finally, better theory is needed to understand the relationships between density, home range size, pair-wise overlap, and total overlap in idealized and wild populations.
